# BE-PIGS: a base-editing tool with deaminases inlaid into Cas9 PI domain significantly expanded the editing scope

**DOI:** 10.1038/s41392-019-0072-7

**Published:** 2019-09-20

**Authors:** Yanhong Wang, Lifang Zhou, Nan Liu, Shaohua Yao

**Affiliations:** State Key Laboratory of Biotherapy and Cancer Center, West China Hospital, Sichuan University, 610041 Chengdu, People’s Republic of China

**Keywords:** Molecular biology, Genetic techniques

**Dear Editor,**

The discovery of base-editing tools sheds new light on both basic biomedical research and clinical therapeutics.^1,2^ Although showing profound performance in a wide range of applications, current base editors have their limitations. In particular, the narrow editing windows of these base editors have significantly limited their targetable sites. In current base editors, the most frequent way to tether deaminase is to fuse it to the N-terminus of the Cas9 protein through flexible peptide linkers. Base editors derived from *Streptococcus pyogenes Cas9* (SpyCas9) or its variants can efficiently edit desired bases in an ~5 nt wide window within the detached,non-target strand (NTS). Maximum editing typically occurs around positions 5–7, counting from the 5′ end of the protospacer, which is about 15 nt away from the protospacer adjacent motif (PAM).^1,3^ Bases outside this narrow window are poorly edited. Given that there are very limited choices for PAMs, many targets were not able to be targeted by current base editors due to the lack of well-positioned PAMs.

To overcome this obstacle, we designed a novel editing system based on the position information of the Cas9/DNA/sgRNA complex. Recently, a cryo-electron microscopy structure of the Cas9/DNA/sgRNA complex was resolved, which for the first time presented the scenario with detached with the NTS included.^4^ According to this structure, parts of PI domain and RuvC3 domain lie just adjacent to and on the same side as the NTS. The C-terminus of the Cas9 protein lies to the other side, opposite to the NTS, and the N-terminus lies between the PI domain and the C-terminus. Consistent with the position information revealed by this structure, fusion of cytosine deaminases, such as APOBEC1, to the N-terminus but not to the C-terminus, with finite linkers, possesses the ability to deaminate cytosine within the NTS. Similarly, lengthening the linker that connected the deaminase and Cas9 protein increased both the editing efficiency and the width of editing window, suggesting that a relatively short distance between deaminases and the NTS yielded a high editing efficiency and wide editing window.^5,6^ Therefore, we hypothesized that inlaying deaminases directly into the PI or RuvC3 domain without interrupting their activities would increase the editing efficiency and broaden the editing window.

To test our hypothesis, we analyzed the secondary structure of this region for proper inlay sites. We found two candidate sites that were located in unstructured loops within the RuvC and PI domains (Fig. 1a), that is, site 1054 (between G1054 and E1055) and site 1246 (between G1246 and S1247), which we named BE-RuvCGE and BE-PIGS, respectively. We inserted APOBEC1 into these two sites using a pair of flexible linkers (Fig. 1b). Transfection of these novel editors together with a previously reported sgRNA into mammalian cells revealed that BE-PIGS induced robust C-to-T base editing while BE-RuvCGE caused mild editing (Fig. 1c, d). This observation was further confirmed with additional two sgRNAs (Fig. S1). On average, BE-PIGS exhibited nearly two-fold greater editing activity than BE- RuvCGE (23.67% vs 12.67%). Therefore, we focused on the BE- PIGS base editor for further characterization.

**Fig. 1:**
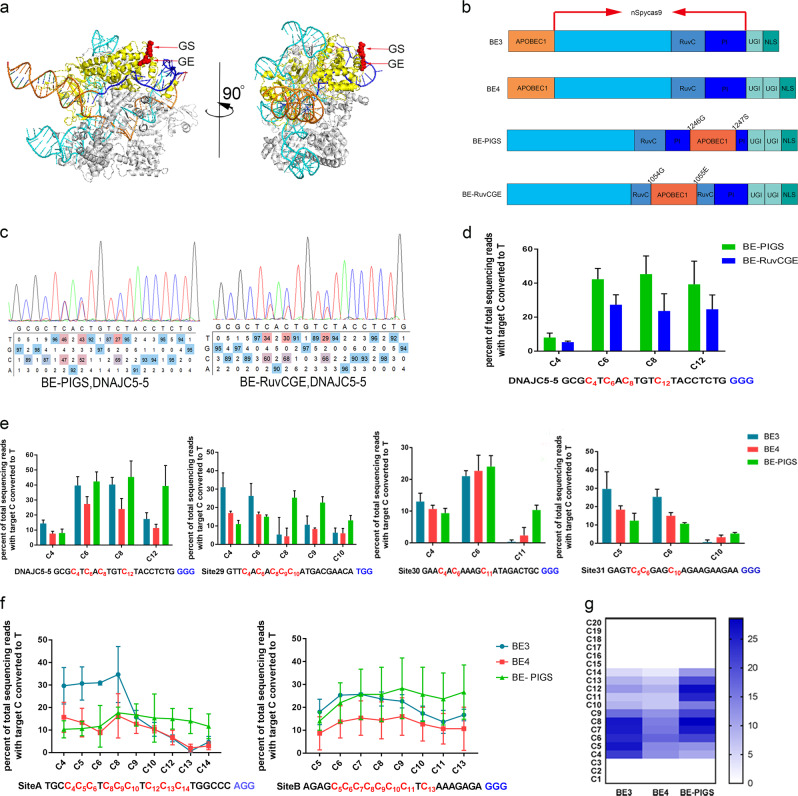
Inlaying APOBEC1 into Cas9 PI domain significantly expanded the editing scope. **a** Cartoon representations of the crystal structure of SpyCas9 with DNA and sgRNA complex (PDB 5Y36). The amino acids G1054, E1055, G1246 and S1247 of SpyCas9 are colored red and shown as spheres. **b** Cartoon representations showing the architectures of BE3, BE-PIGS and BE-RuvCGE. **c** Sanger sequencing and EditR analysis of C-to-T base editing in target DNAJC5-5 by BE-PIGS and BE-RuvCGE. **d** Quantification of base editing efficiencies of each target C in DNAJC5-5. The protospacer and PAM sequences of DNAJC5-5 are shown below the quantification, with target Cs shown in red and PAM shown in blue. **e**, **f** Comparison of base editing induced by BE-PIGS and by BE3 or BE4. The protospacer and PAM sequences of each target site are shown below the quantification, with target Cs shown in red and PAM shown in blue. **g** Heat maps showing the average editing efficiency of BE3, BE4, and BE-PIGS at each position across six sites, each of which was independently repeated three times

We compared the editing efficiency of BE-PIGS to that of BE3 and BE4, two of the most popular and efficient base editors.^7^ BE-PIGS showed a slightly low editing efficiency than BE3 and BE4 in the cytosines (Cs) located within the editing window of BE3 or BE4 (positions 4–8, numbering the PAM as positions 21–23), while performing much higher editing in Cs downstream of this window (Fig. 1e), indicating that the BE-PIGS base editor might have a wider editing window than that of BE3 or BE4. To further confirm this, we characterized the editing window of BE-PIGS with additional targets that possess multiple Cs. As shown in Fig. 1f, BE-PIGS achieved considerable editing in Cs that were only six bases away from the PAM (position 14). Together, these data demonstrated that inlaying APOBEC1 into the PI domain significantly increased the editing width (Fig. 1g), ranging from position 4 to position 14, with the maximum editing occurring around position 7–13 (Fig. 1g). This finding was in consistent with a recent publication in which tethering deaminase to the PI domain of a circular permuted Cas9 also expanded the editing window.^8^

Then, we examined the effects of the length of linker, connecting the deaminase and the Cas9 PI domain on the editing efficiency and window. We first shortened the N-terminal linker, keeping the C-terminal linker unchanged. We showed that shortening the N-terminal linker from 16 aa to 8 aa or 3 aa generally reduced the editing efficiency in all targetable Cs, but did not obviously change the width of the editing window. Similar effects were also observed when the C-terminal linker was shortened from 32 aa to 20 aa or 5 aa (Fig. S3).

Recently, a BE system based on Sun-tag (GCN4/scFv) mediated linkage of deaminase and Cas9 was shown to expand the editing window (BE-PLUS).^9^ To test whether the Sun-tag system is also feasible for and further expands the editing window of BE-PIGS, we inserted six copies of GCN4 helices into the PIGS site to form PIGS-GCN4. We showed that transfections of this construct together with scFv tagged APOBEC1 yielded a slight reduction in editing in a set of target sites, and did not further enlarge the editing window (Fig. S4).

Previous studies have revealed that truncating the spacers within SpyCas9 sgRNAs reduced off-target effects and altered the editing window in SpyCas9 derived base editors,^6,10^ we therefore tested the effects of sgRNA truncation on our base-editing system. We truncated the spacers within a set of sgRNAs from 20 to 17 or 18 nt. In one out of the three target sites tested, the truncated guide RNA led to a narrowed editing window (Fig. S5). However, this phenomenon was not observed in the rest target sites (Fig. S5), suggesting that the effects of sgRNA truncation on the editing window are protospacer specific. In summary, we generated a novel base-editing system by inlaying deaminase into the PI domain of the SpyCas9 protein, BE-PIGS. We showed that internally inlayed deaminase or GCN4 tag retained their ability to induce base substitution but did not destroy Cas9 activity for target recognition. BE-PIGS has a much wider editing window than BE3 or BE4, which is particularly useful in cases where wide editing ranges are required or targeted bases are extremely close to the PAM.

## Supplementary information

Supplementary information.
