# Supplementary material for: BE-PIGS: a base-editing tool with deaminases inlaid into Cas9 PI domain significantly expanded the editing scope
Source: Signal Transduct Target Ther. 2019 Sep 20;4:36. doi: 10.1038/s41392-019-0072-7 (PMC6799832; doi:10.1038/s41392-019-0072-7)
Supplement: Supplementary file 1 — Supplementary information. [file 41392_2019_72_MOESM1_ESM.doc]

**Supplementary information

Table of Contents:**

**Supplementary information, Figures and Tables**

Fig. S1. BE-PIGS is superior to BE-RuvCGE.

Fig. S2. Representative Sanger sequencing results and EditR analysis for (Figure 1e-1f).
Fig. S3. The effect of the linker length between Cas9 and APOBEC1 on the editing efficiency.

Fig. S4. Base editing activity of BE-PIGS-GCN4.
Fig. S5. Effects of sgRNA truncation on editing efficiency of BE-PIGS.
Table S1. List of the base editor targets used in this study.

Table S2. Summary of primers for amplification of each target sites.

**Supplementary information, Materials and Methods**

**Supplementary information, References**


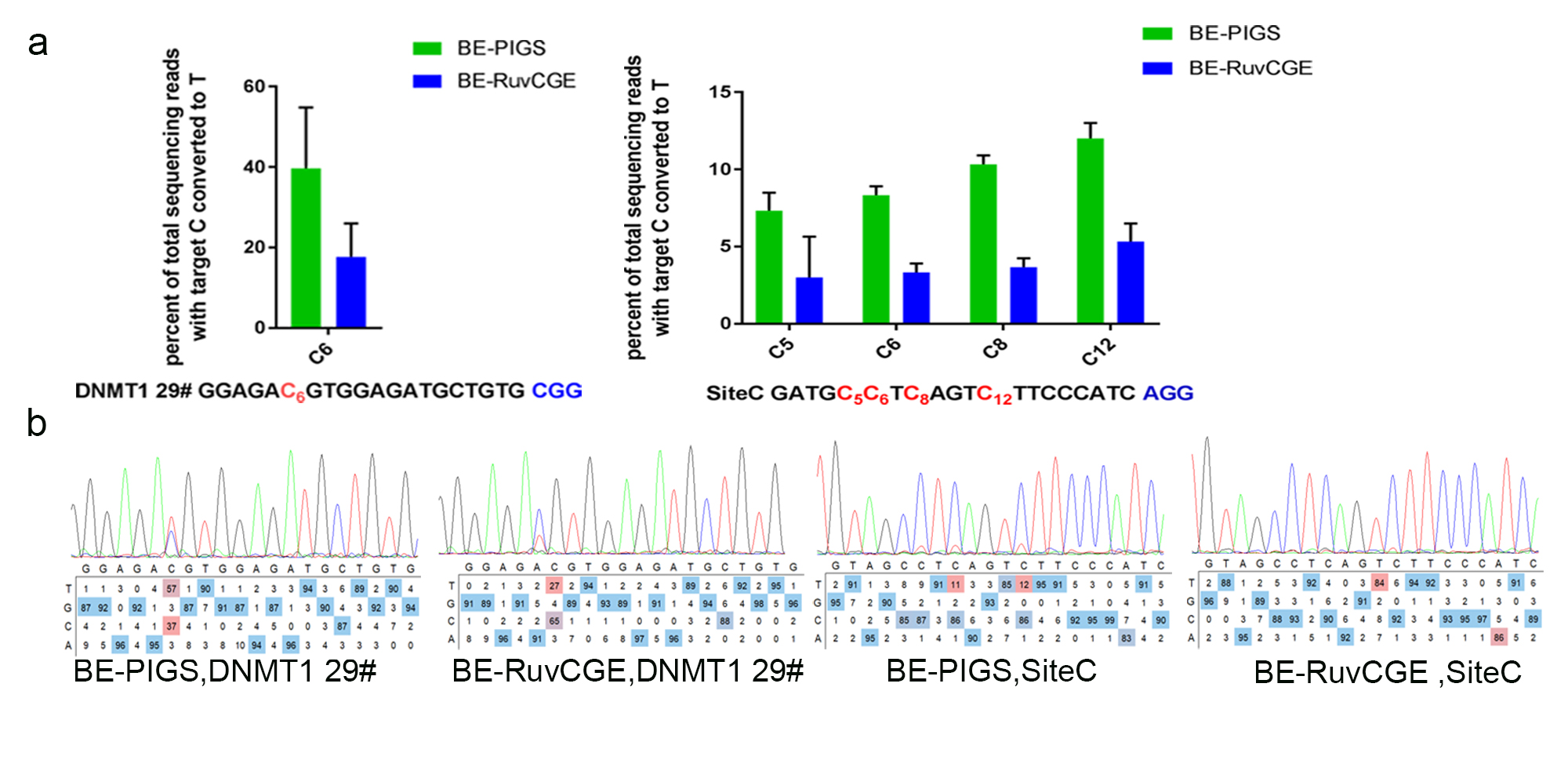


**Supplementary information, Fig. S1** BE-PIGS is superior to BE-RuvCGE

**a** Quantitative analysis of C to T editing efficiency of BE-PIGS and BE-RuvCGE; **b** Representative Sanger sequencing results and EditR analysis for (a)**.**


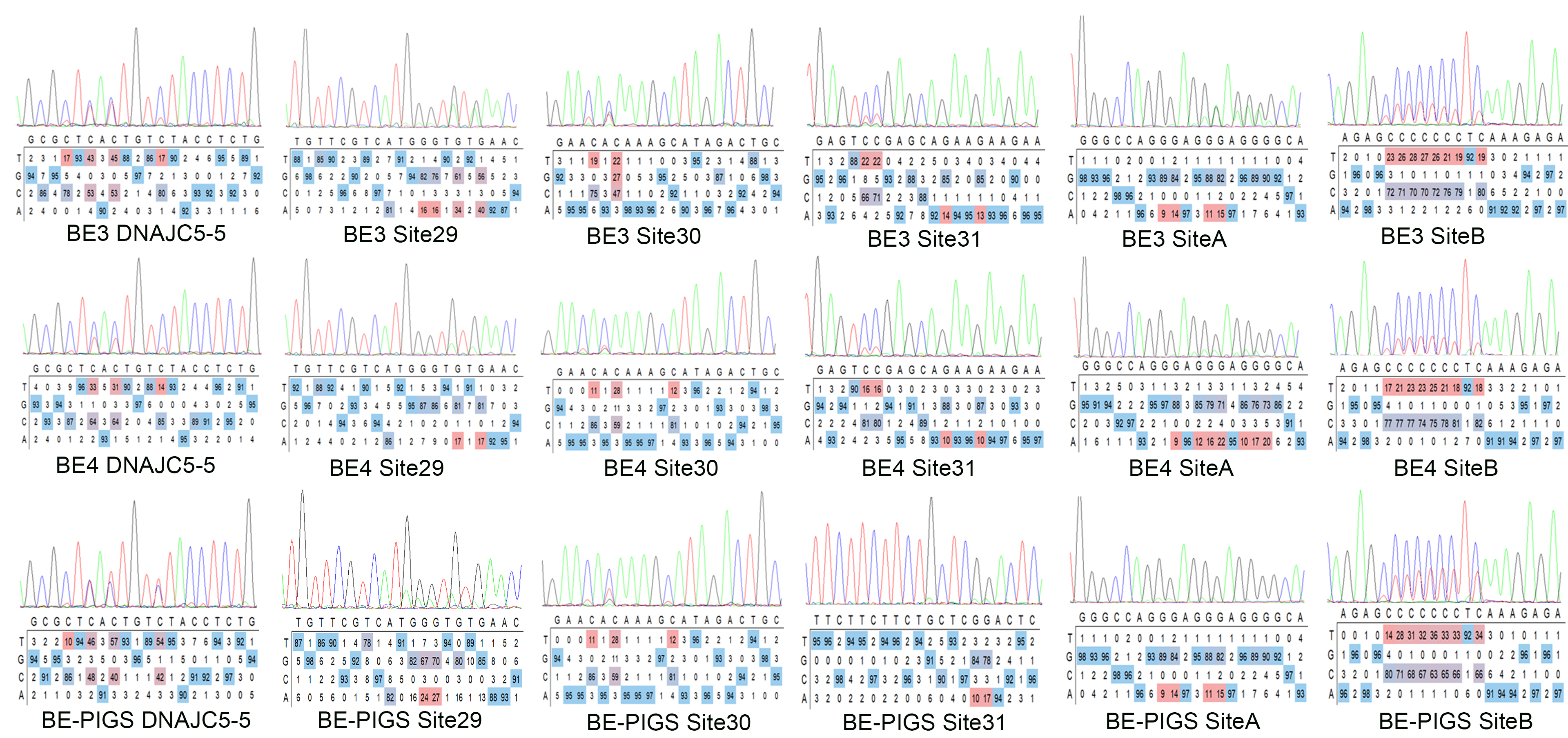
**Supplementary information, Fig. S2** Representative Sanger sequencing results and EditR analysis for (Figure 1e-1f).


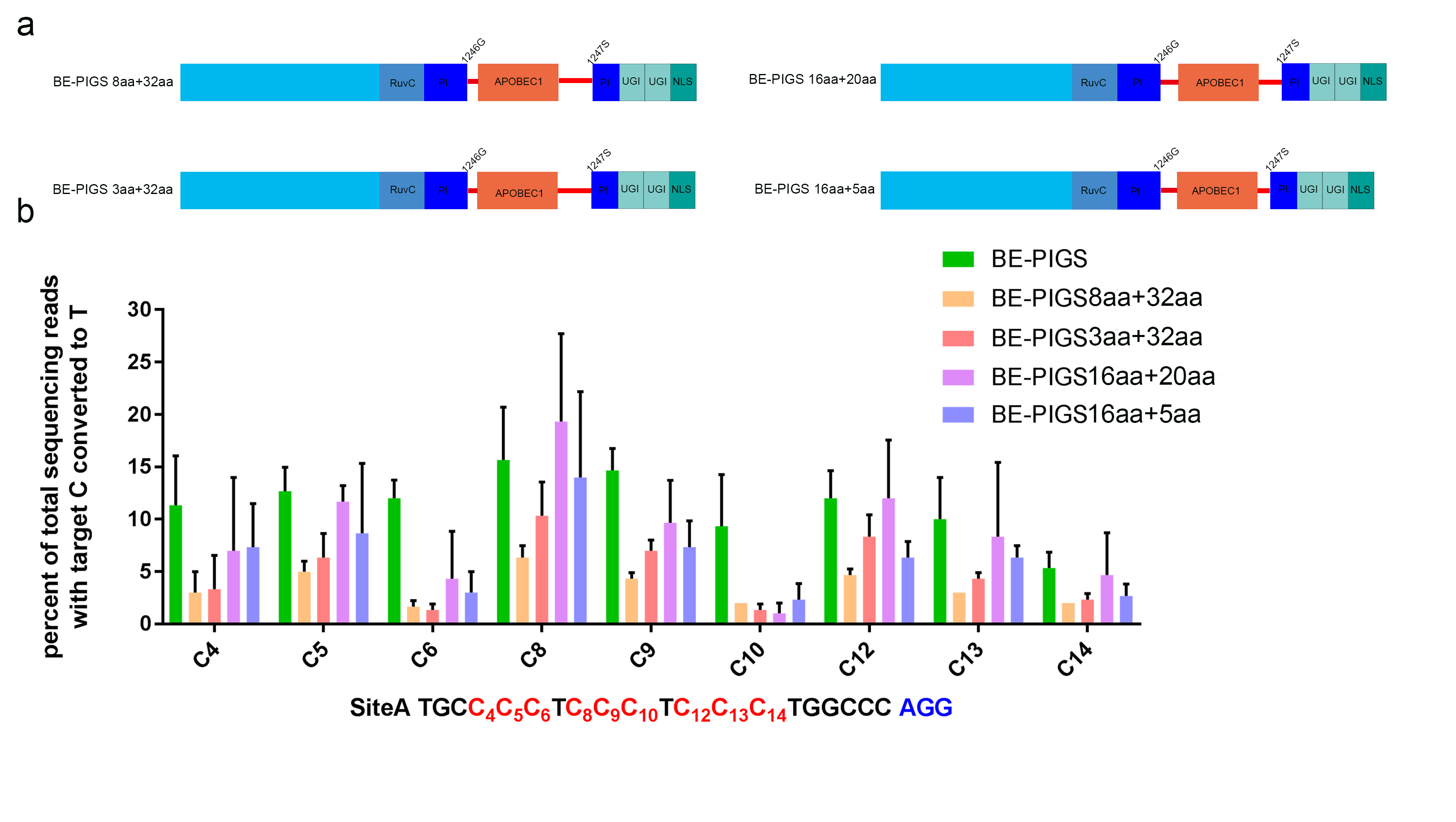


**Supplementary information, Fig. S3** The effect of the linker length between Cas9 and APOBEC1 on the editing efficiency.

**a.** The architectures of BE-PIGSs with variant linkers. **b.** Comparison of base editing induced by original BE-PIGS and by its derivations.


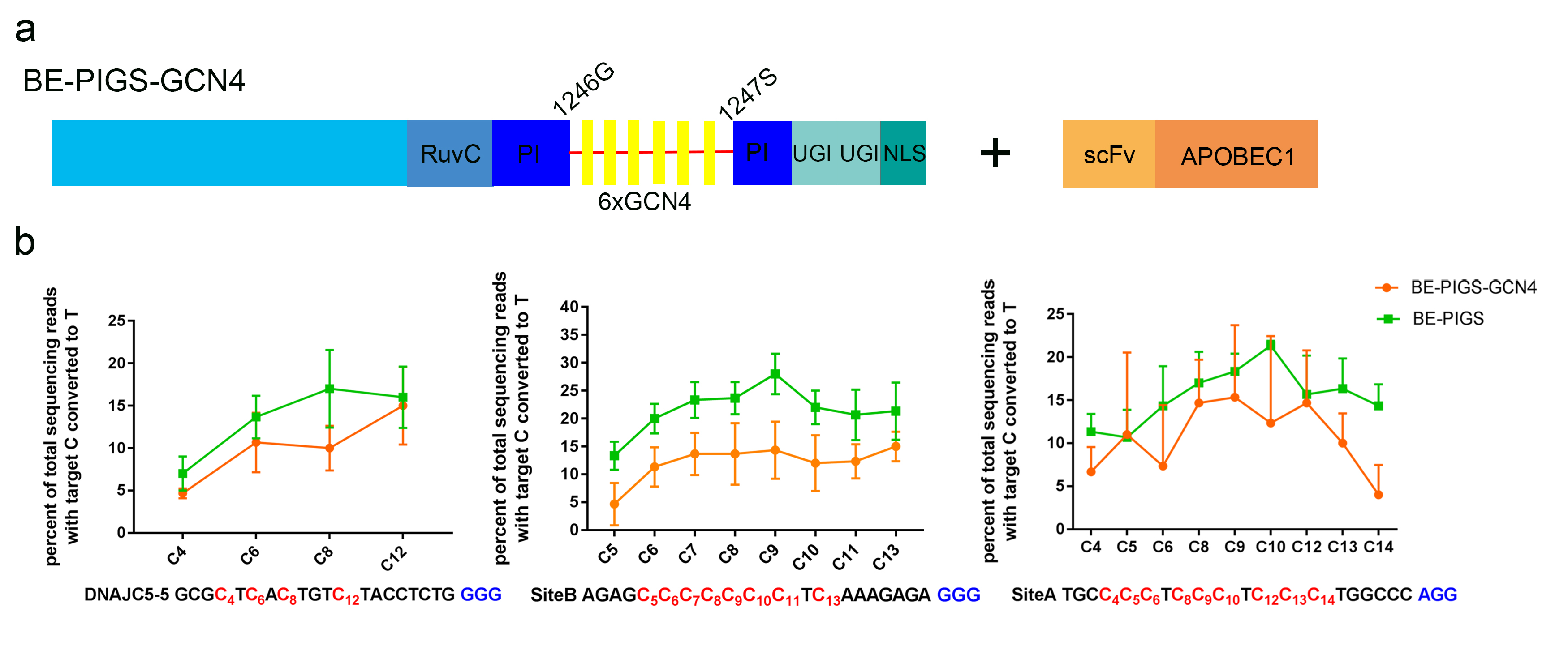


**Supplementary information, Fig. S**4 Base editing activity of BE-PIGS-GCN4.

**a.** Architecture of BE-PIGS-GCN4. **b**. Base editing efficiency of BE-PIGS and BE-PIGS-GCN4.

**
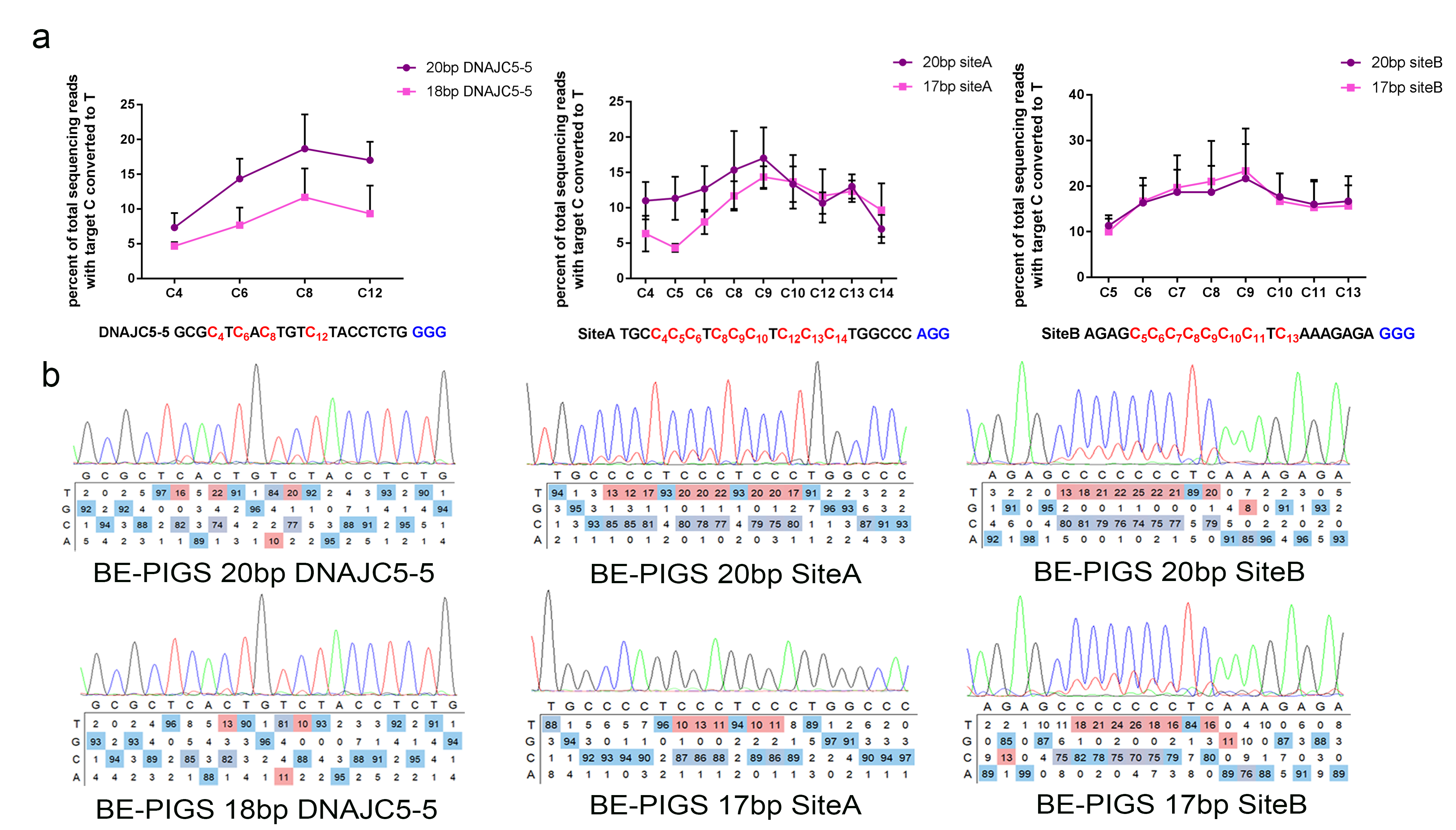
**

**Supplementary information, Figure. S5.** Effects of sgRNA truncation on editing efficiency of BE-PIGS.

**a.** Quantitative analysis of C to T editing efficiency of BE-PIGS coupled with full-length or truncated sgRNAs. The truncated protospacer sequences were showed in Supplementary Table 1. **b.** Representative Sanger sequencing results and EditR analysis for (a).

**Table S1.** **List of the targets tested in this study**

| sgRNA | Target sequence | Oligo-F | Oligo-R | Reference |
| --- | --- | --- | --- | --- |
| DNAJC5-5 | GCGCTCACTGTCTACCTCTG **GGG** | CACCGCGCTCACTGTCTACCTCTG | AAACCAGAGGTAGACAGTGAGCGC | 1 |
| 18bpDNAJC5-5 | GCTCACTGTCTACCTCTG **GGG** | CACCGCTCACTGTCTACCTCTG | AAACCAGAGGTAGACAGTGAGC | 1 |
| site C | GTAGCCTCAGTCTTCCCATC **AGG** | CACCGTAGCCTCAGTCTTCCCATC | AAACGATGGGAAGACTGAGGCTAC | This study |
| DNMT1 29# | GGAGACGTGGAGATGCTGTG **CGG** | CACCGGAGACGTGGAGATGCTGTG | AAACCACAGCATCTCCACGTCTCC | 2 |
| site A | TGCCCCTCCCTCCCTGGCCC **AGG** | CACCGTGCCCCTCCCTCCCTGGCCC | AAACGGGCCAGGGAGGGAGGGGCAC | 3 |
| 17bp siteA | CCCTCCCTCCCTGGCCC **AGG** | CACCGCCCTCCCTCCCTGGCCC | AAACGGGCCAGGGAGGGAGG | 3 |
| site B | AGAGCCCCCCCTCAAAGAGA **GGG** | CACCGAGAGCCCCCCCTCAAAGAGA | AAACTCTCTTTGAGGGGGGGCTCTC | 3 |
| 17bp siteB | GCCCCCCCTCAAAGAGA **GGG** | CACCGCCCCCCCTCAAAGAGA | AAACTCTCTTTGAGGGGGGGCC | 3 |
| site 29 | GTTCACACCCATGACGAACA **TGG** | CACCGTTCACACCCATGACGAACA | AAACTGTTCGTCATGGGTGTGAAC | 4 |
| site 30 | GAACACAAAGCATAGACTGC **GGG** | CACCGAACACAAAGCATAGACTGC | AAACGCAGTCTATGCTTTGTGTTC | 4 |
| site 31 | GAGTCCGAGCAGAAGAAGAA **GGG** | CACCGAGTCCGAGCAGAAGAAGAA | AAACTTCTTCTTCTGCTCGGACTC | 4 |

The PAM motif in each target sequence was shown in bold. Oligo-F and Oligo-R were used to clone sgRNA expression vector.

| Target site | | chromosome | Forward primer | Reverse primer |
| --- | --- | --- | --- | --- |
| DNAJC5-5 | | Chr20 | TCTGTCTGTGCACGTGGCAA | AGCTGTGACCAGTTCAACGC |
| 18bpDNAJC5-5 | | Chr20 | TCTGTCTGTGCACGTGGCAA | AGCTGTGACCAGTTCAACGC |
| site C | Chr2 | | GCTCTGGACCCTTTATTTGA | CTTGTCCCTCTGTCAATGGC |
| DNMT1 29# | Chr12 | | AAGCATGGCTTCGTGCTTGA | TGGAGTAGGTGCGCGAATTG |
| site A | Chr 2 | | GCTCTGGACCCTTTATTTGA | CTTGTCCCTCTGTCAATGGC |
| 17bp siteA | Chr2 | | GCTCTGGACCCTTTATTTGA | CTTGTCCCTCTGTCAATGGC |
| site B | Chr20 | | GCCTGGAGGGAAATCTTAGG | GGGCTTCACTGAGTCTCCAC |
| 17bp siteB | Chr20 | | GCCTGGAGGGAAATCTTAGG | GGGCTTCACTGAGTCTCCAC |
| site 29 | Chr12 | | AAATCAAGTGGGGCGATGCT | GAAAGCCAGTCCCCAGAACC |
| site 30 | Chr5 | | ACAGGCTACCCCCTAAGT | TCCCAAGTGAGAAGCCAGTG |
| site 31 | Chr2 | | GCTCTGGACCCTTTATTTGA | CTTGTCCCTCTGTCAATGGC |

**Table S2.**  **Summary of primers for amplification of each target sites**

**Supplementary information, Materials and Methods**

**Plasmid construction**BE3 and BE4 plasmids were obtained from addgene (#73021 and #100802). BE-plus plasmids were kind gifts from Dr. Xingxu Huang. Seamless cloning was performed to construct base editor plasmids. Briefly, BE4 plamid was used as a parent plasmid to generate BE-PIGS, BE-PIGS-GCN4 or BE- RuvCGE. APOBEC1 or GCN4 with variant linkers were PCR amplified and cloned into the BE4 derived fragment with N-terminal APOBEC1 deleted. sgRNA expression vectors were constructed by ligating annealed oligonucleotide duplexes into pU6 sgRNA cut with Bbs1. Oligos used to generate sgRNA expression plasmids were listed in supplementary Table 1. All plasmids were verified by Sanger sequencing.

**Cell culture and transfection**

HEK 293T were maintained in Dulbecco's modified Eagles's medium (Thermo Fisher Scientific), supplemented with 10%(v/v) fetal bovine serum (life technologies),1% pencillin/streptomycin, at 37℃ with 5% CO2.HEK 293T were transfected with TranseasyTM (Forgene) according to manufacturer’s instruction. Puromycin selection was performed using media containing 4ug/ml of puro-mycin.72 hours post transfection, genomic DNA was extracted for further analyasis.

**Base editing analysis with EditR software**

On-target genomic regions of interest were amplified by PCR and then were analyzed with Sanger sequencing. The primers used for amplifying each target loci were listed in Supplementary table 2. The Sanger sequencing graphs were further quantified by EditR software (baseditr.com), according to the author’s description^5^ .

**Supplementary information, References:**

1 Yao, X. *et al.* Gene Therapy of Adult Neuronal Ceroid Lipofuscinoses with CRISPR/Cas9 in Zebrafish. *Hum Gene Ther* **28**, 588-597, doi:10.1089/hum.2016.190 (2017).

2 He, Z. Y. *et al.* In Vivo Ovarian Cancer Gene Therapy Using CRISPR-Cas9. *Hum Gene Ther* **29**, 223-233, doi:10.1089/hum.2017.209 (2018).

3 Kim, Y. B. *et al.* Increasing the genome-targeting scope and precision of base editing with engineered Cas9-cytidine deaminase fusions. *Nat Biotechnol* **35**, 371-376, doi:10.1038/nbt.3803 (2017).

4 Huang, T. P. *et al.* Author Correction: Circularly permuted and PAM-modified Cas9 variants broaden the targeting scope of base editors. *Nat Biotechnol* **37**, 820, doi:10.1038/s41587-019-0168-1 (2019).

5 Kluesner, M. G. *et al.* EditR: A Method to Quantify Base Editing from Sanger Sequencing. *CRISPR J* **1**, 239-250, doi:10.1089/crispr.2018.0014 (2018).
